# Supplementary material for: Dopamine Buffering Capacity Imaging: A Pharmacodynamic fMRI Method for Staging Parkinson Disease
Source: Front Neurol. 2020 May 6;11:370. doi: 10.3389/fneur.2020.00370 (PMC7232584; doi:10.3389/fneur.2020.00370)
Supplement: Supplementary file 1 [file Table_1.DOCX]

Supplementary Material

# Supplementary Data

All of the figures and tables below are based on 100 iterations of added Gaussian noise.

# Supplementary Figures and Tables

## Supplementary Figures


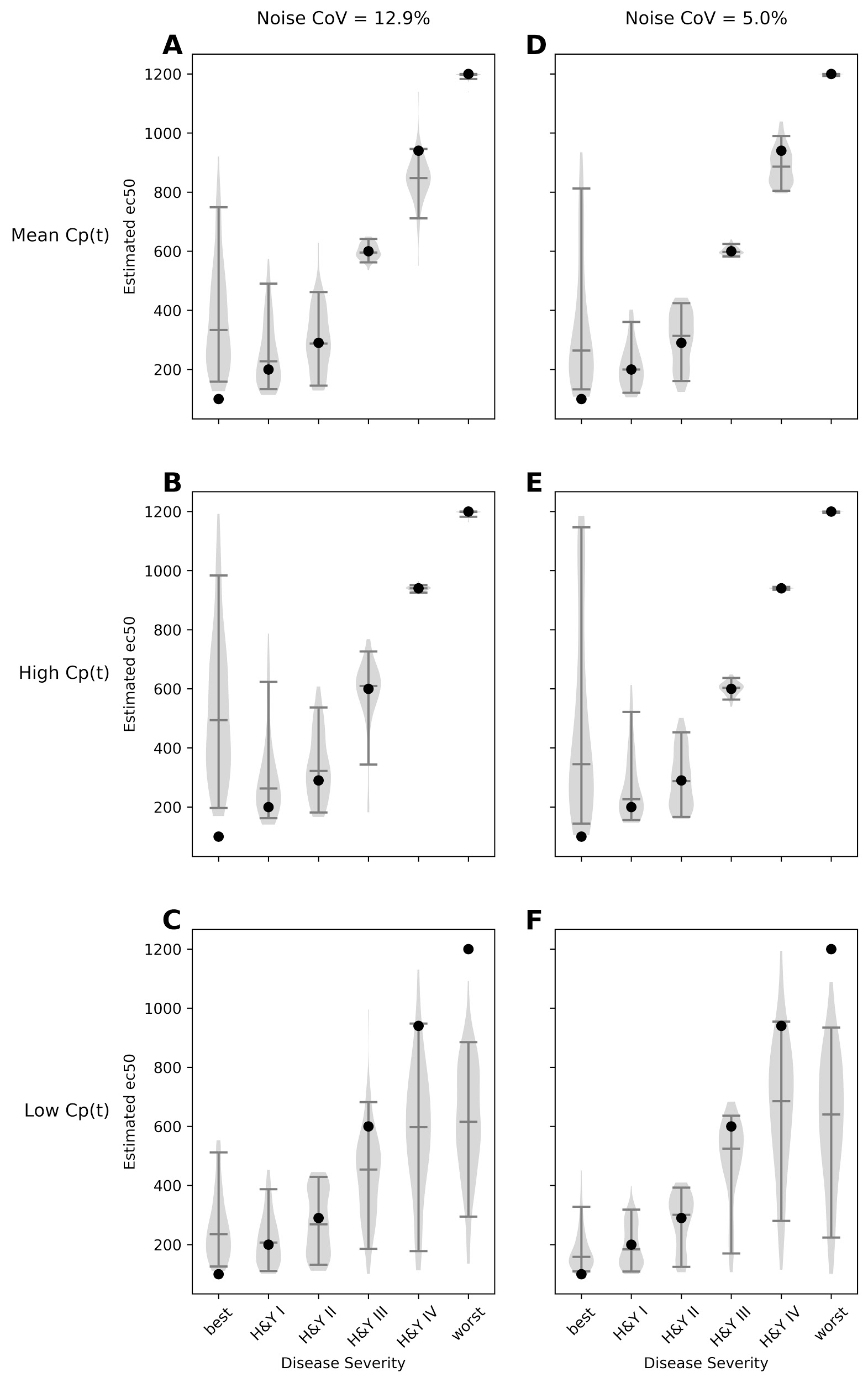


**Supplementary Figure 1. Accuracy of *EC*_50_, noiseCoV = 12.9% or 5%.** Width of plot is proportional to frequency of output of the given magnitude. Filled circle: input *EC*_50_. Horizontal lines note the 5th, 50th and 95th percentiles.


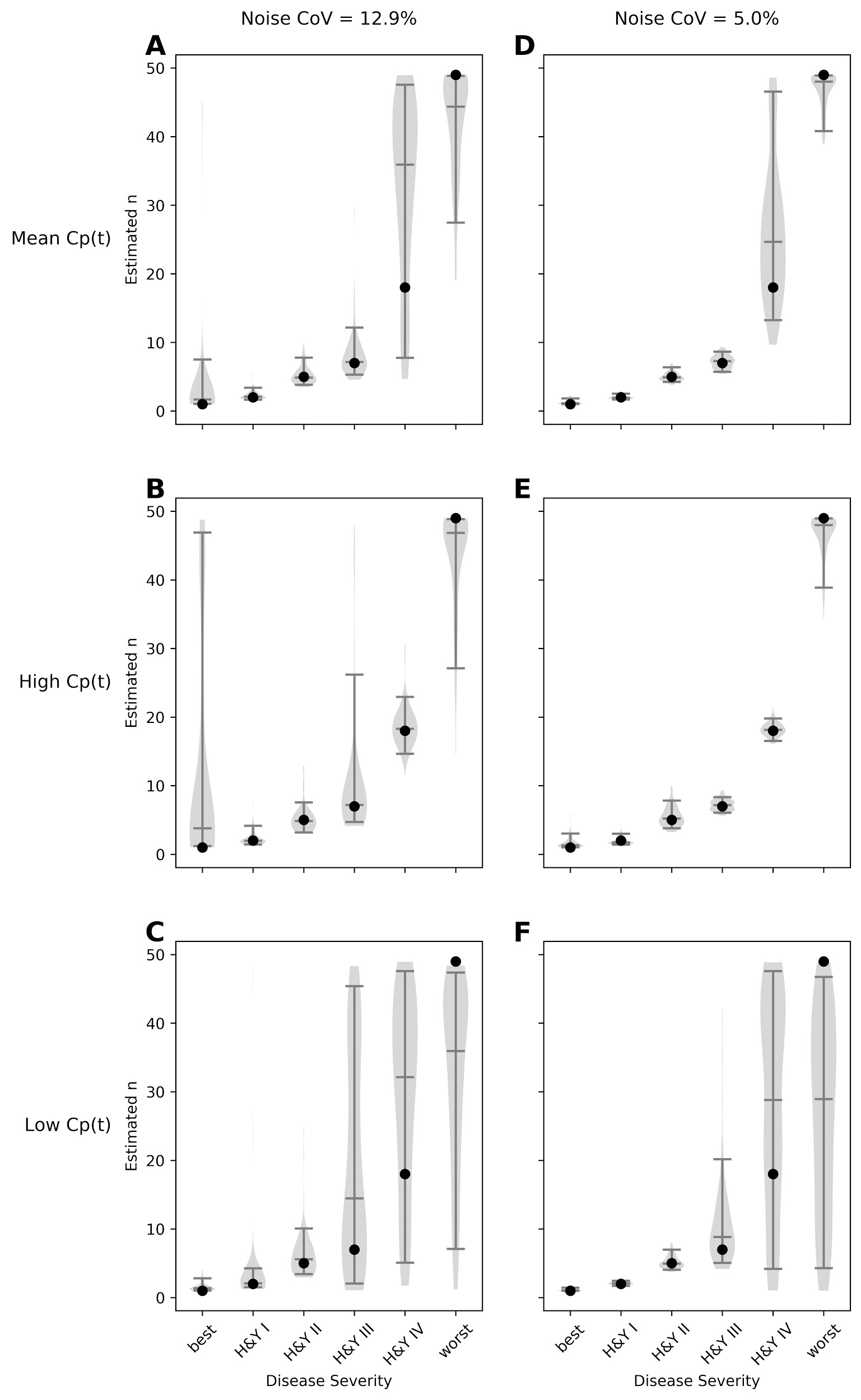


**Supplementary Figure 2. Accuracy of *n*, noiseCoV = 12.9% or 5%.** Width of plot is proportional to frequency of output of the given magnitude. Filled circle: input *n*. Horizontal lines note the 5th, 50th and 95th percentiles.


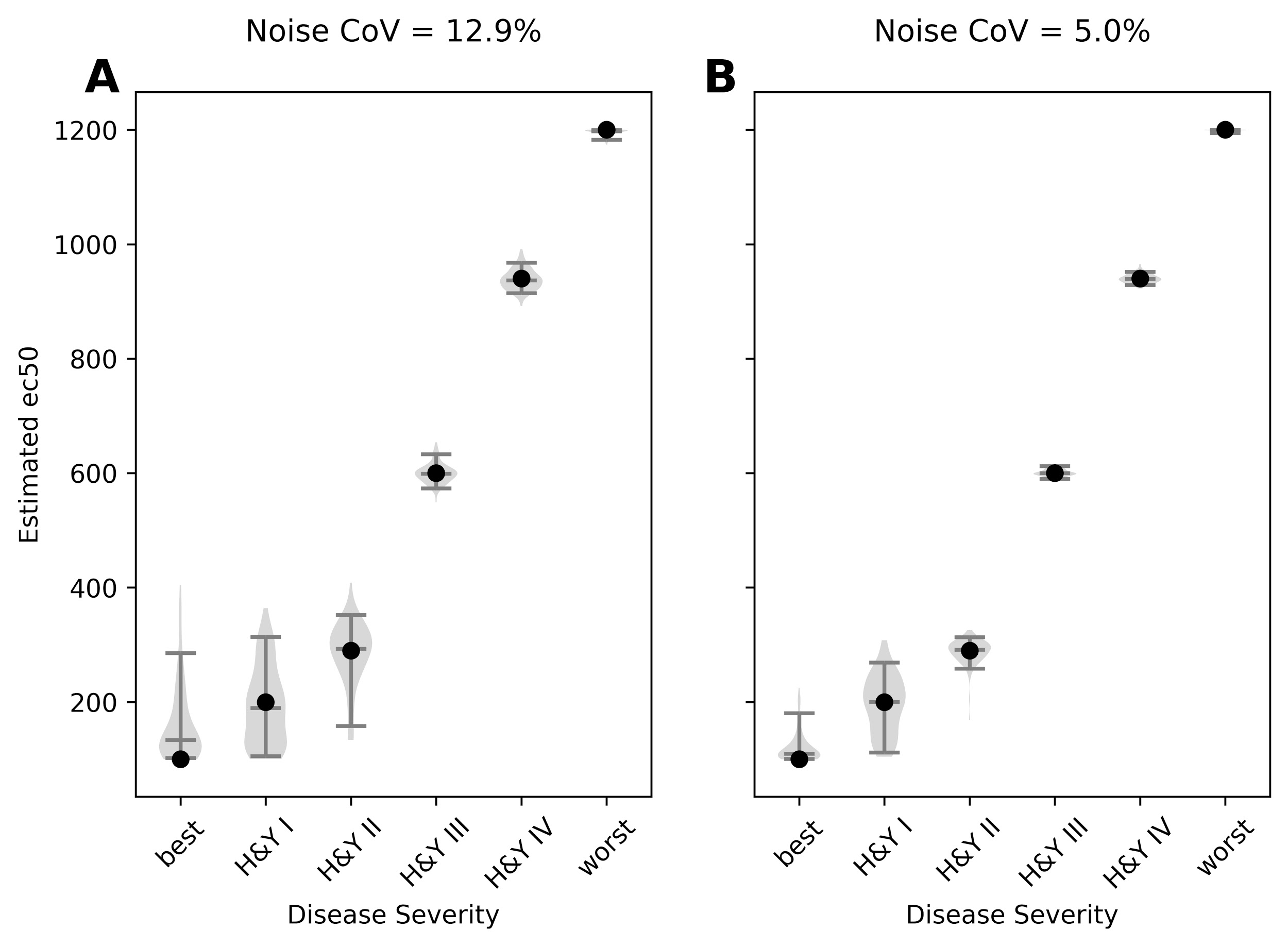


**Supplementary Figure 3. Accuracy of *EC*_50_, “slow” concentration curve.** Width of plot is proportional to frequency of output of the given magnitude. Filled circle: input *EC*_50_. Horizontal lines note the 5th, 50th and 95th percentiles.


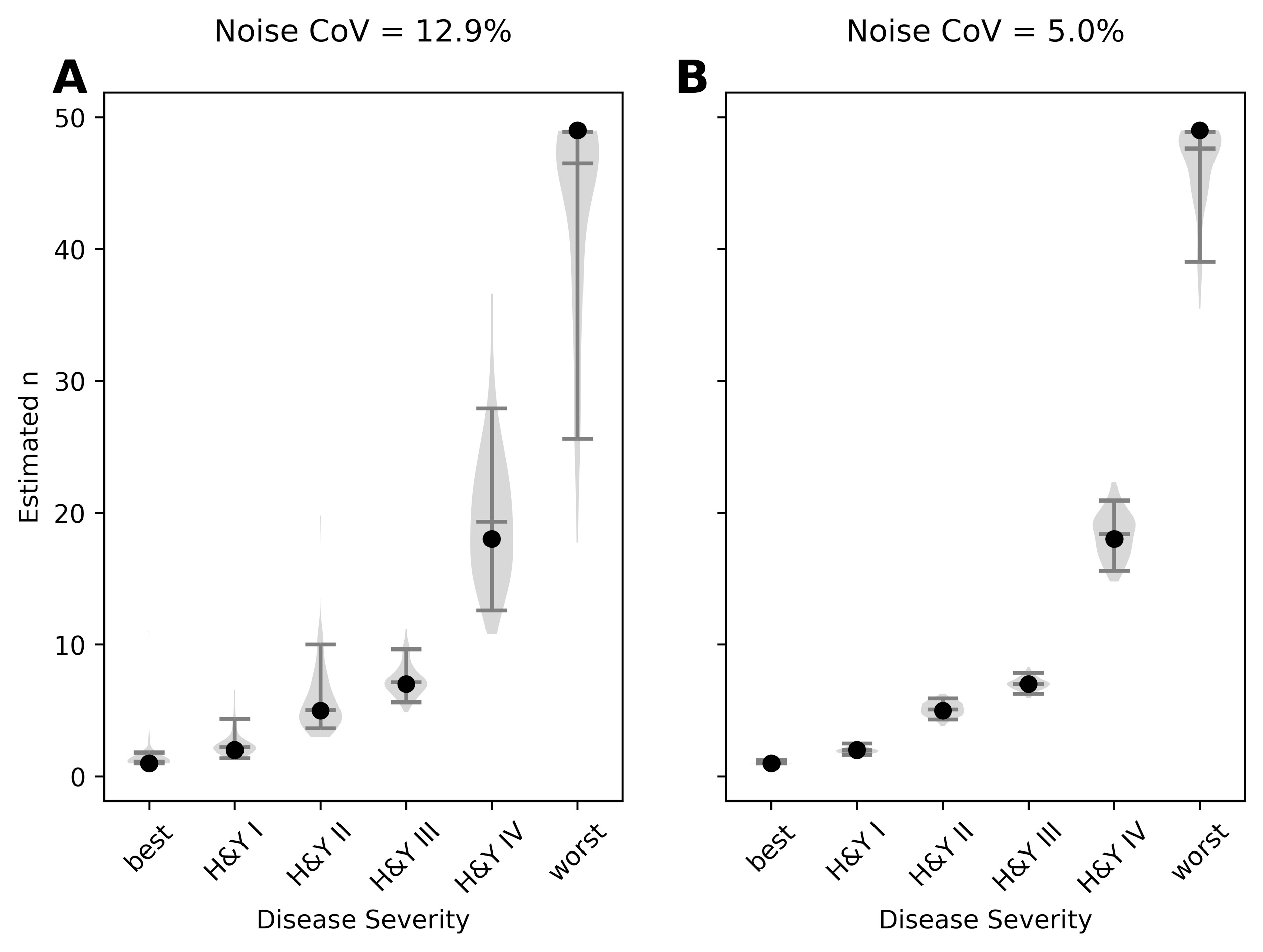


**Supplementary Figure 4. Accuracy of *n*, “slow” concentration curve.** Width of plot is proportional to frequency of output of the given magnitude. Filled circle: input EC_50_. Horizontal lines note the 5th, 50th and 95th percentiles.

## 2.2 Supplementary Tables

| **H&Y** | **Cp** | **target** | **median** | **5th %tile** | **95th %tile** |
| --- | --- | --- | --- | --- | --- |
| best | mean | 0.0025 | 0.0068 | 0.0034 | 0.0173 |
| I | mean | 0.0052 | 0.0059 | 0.0030 | 0.0131 |
| II | mean | 0.0089 | 0.0091 | 0.0033 | 0.0152 |
| III | mean | 0.0248 | 0.0247 | 0.0231 | 0.0269 |
| IV | mean | 0.0347 | 0.0349 | 0.0291 | 0.0747 |
| worst | mean | 0.1386 | 0.1374 | 0.1317 | 0.1385 |
| best | hi | 0.0025 | 0.0089 | 0.0033 | 0.0358 |
| I | hi | 0.0052 | 0.0069 | 0.0040 | 0.0129 |
| II | hi | 0.0089 | 0.0105 | 0.0038 | 0.0196 |
| III | hi | 0.0248 | 0.0251 | 0.0119 | 0.0306 |
| IV | hi | 0.0347 | 0.0346 | 0.0334 | 0.0363 |
| worst | hi | 0.1386 | 0.1381 | 0.1321 | 0.1386 |
| best | lo | 0.0025 | 0.0074 | 0.0029 | 0.0175 |
| I | lo | 0.0052 | 0.0054 | 0.0027 | 0.0146 |
| II | lo | 0.0089 | 0.0081 | 0.0031 | 0.0167 |
| III | lo | 0.0248 | 0.0230 | 0.0069 | 0.0296 |
| IV | lo | 0.0347 | 0.0529 | 0.0099 | 0.1357 |
| worst | lo | 0.1386 | 0.0656 | 0.0099 | 0.1333 |

**Supplementary Table 1. Accuracy of *k_e_*, noise CoV = 12.9%.** “Mean,” “hi,” and “lo” are the three time–activity curves described in the Pharmacokinetics section of Methods. Roman numerals refer to the mean pharmacodynamic values from the Hoehn and Yahr categories in [M Contin et al., 2001](http://f1000.com/work/citation?ids=6099309&pre=&suf=&sa=0). “Best” and “worst” refer to the extreme pharmacodynamic values from the same reference.

| **H&Y** | **Cp** | **target** | **median** | **5th %tile** | **95th %tile** |
| --- | --- | --- | --- | --- | --- |
| best | mean | 0.0025 | 0.0046 | 0.0029 | 0.0100 |
| I | mean | 0.0052 | 0.0052 | 0.0029 | 0.0093 |
| II | mean | 0.0089 | 0.0097 | 0.0039 | 0.0137 |
| III | mean | 0.0248 | 0.0248 | 0.0241 | 0.0256 |
| IV | mean | 0.0347 | 0.0350 | 0.0325 | 0.0380 |
| worst | mean | 0.1386 | 0.1385 | 0.1364 | 0.1386 |
| best | hi | 0.0025 | 0.0047 | 0.0029 | 0.0101 |
| I | hi | 0.0052 | 0.0061 | 0.0030 | 0.0115 |
| II | hi | 0.0089 | 0.0087 | 0.0030 | 0.0168 |
| III | hi | 0.0248 | 0.0249 | 0.0232 | 0.0265 |
| IV | hi | 0.0347 | 0.0348 | 0.0343 | 0.0353 |
| worst | hi | 0.1386 | 0.1385 | 0.1363 | 0.1386 |
| best | lo | 0.0025 | 0.0039 | 0.0028 | 0.0074 |
| I | lo | 0.0052 | 0.0047 | 0.0028 | 0.0092 |
| II | lo | 0.0089 | 0.0093 | 0.0031 | 0.0140 |
| III | lo | 0.0248 | 0.0245 | 0.0073 | 0.0264 |
| IV | lo | 0.0347 | 0.0587 | 0.0059 | 0.1293 |
| worst | lo | 0.1386 | 0.0492 | 0.0086 | 0.1346 |

**Supplementary Table 2. Accuracy of *k_e_*, noise CoV = 5%.** “Mean,” “hi,” and “lo” are the three time–activity curves described in the Pharmacokinetics section of Methods. Roman numerals refer to the mean pharmacodynamic values from the Hoehn and Yahr categories in [M Contin et al., 2001](http://f1000.com/work/citation?ids=6099309&pre=&suf=&sa=0). “Best” and “worst” refer to the extreme pharmacodynamic values from the same reference.
